# A method of constructing a dynamic chart depth model for coastal areas

**DOI:** 10.7717/peerj.15616

**Published:** 2023-07-20

**Authors:** Minglei Guan, Chenyang Tian, Bin Wang, Fangzheng Ji, Rui Sun, Song Yu, Chongping Wang, Qi Wang, Jingzhe Wang, Wei Zhang, Dejin Zhang

**Affiliations:** 1 Institute of Applied Artificial Intelligence of the Guangdong-Hong Kong-Macao Greater Bay Area, Shenzhen Polytechnic, Shenzhen, Guangdong, China; 2 School of Artificial Intelligence, Shenzhen Polytechnic, Shenzhen, Guangdong, China; 3 Guangdong Key Laboratory of Urban Informatics, Shenzhen University, Shenzhen, Guangdong, China; 4 School of Marine Technology and Geomatics, Jiangsu Ocean University, Lianyungang, Jiangsu, China; 5 Institute of Surveying and Mapping, Department of Natural Resources of Guangdong Province, Guangzhou, China

**Keywords:** Dynamic chart depth, Water level correction, Residual water level, Depth datum

## Abstract

The depth is important for vessel navigation at sea. Currently, most vessels use electronic navigation charts to navigate at sea. In coastal areas, especially close to shallow water areas, the dynamic change of the water level is very important to safe navigation. Ships calculate the change of water level by using up-to-date tide tables, to obtain the dynamic water depth in the channels. However, the depth caused by the tide and non-tidal components may reach several meters in some seas, causing the dynamic depth below the safety depth, which can easily lead to grounding of vessels stranding accidents. The channel is regularly dredged to achieve navigational depth. Without regular dredging, the offshore non-channel area becomes the common area of ship grounding. The dynamic chart depth model studied in this article can provide real-time depth, which serves the ships navigation in the non-channel. The model incorporates the chart depth and the dynamic water levels on the same reference datum. The chart depth is from the electronic navigational chart depth. The dynamic water levels are constructed by the simulated tidal levels and continuous series of nontidal residual. We then designed a deviation correction method to reduce the discrepancy of the simulated tidal level with the actual water level, including datum offset correction and residual water level correction. Finally, by merging the revised dynamic water levels with the electronic navigational chart depth, we obtained the dynamic chart depth model of the study region.

## Introduction

The depth of the chart plays a crucial role in sea vessel navigation. In order to navigate safely, ships must take into account the dynamic depth, which is the sum of the chart depth and the dynamic water levels at the time. Chart depth refers to the depth of the seafloor from chart datum. Dynamic water levels, on the other hand, refer to the constantly changing depth of water above the chart datum. To ensure safe navigation and maximize channel utilization rates, the lowest astronomical tide is selected as chart datum. However, in some seas, the depth caused by tidal and non-tidal components can reach several meters, causing the dynamic depth to fall below the chart datum. This can easily lead to accidents such as grounding and stranding, particularly for fishing boats and yachts (Amdahl, Ehlers & Leira, 2013; Zhu et al., 2022). Authorities regularly dredge the channel to reach the charted depth and ensure vessel safety. However, non-channel areas such as shallow water regions in coastal and reef areas are still common sites of ship grounding accidents. The dynamic chart depth studied in this article can provide real-time depth information for ships navigating in non-channel areas. Although there are few tidal observation stations in the area, most of them use tide tables for tidal level information, which only include the tidal range and tidal time difference, and do not contain residual water level information. The residual water level, which can have random and deterministic components, generally refers to the non-tidally forced water level variability. In abnormal weather conditions, the difference between the real water depth and the chart water depth can be several meters, which can easily lead to accidents such as grounding and stranding, especially for fishing boats and yachts (Lee, 2021; Lee et al., 2019; Amdahl, Ehlers & Leira, 2013).

The traditional method of predicting tides involves the use of tide tables which provide information about the daily timings and levels of high and low tides over a long period. To calculate the tide levels at other times, an intermediate tidal curve is used. Obtaining the residual water level for a particular location using a tide gauge signal is a relatively simple task. This involves using the harmonic analysis method to remove the harmonically predicted part from the observed water level data to obtain the residual water level.

A commonly used method to determine water depth is by using tide tables and chart depth. However, obtaining an accurate “nowcast” of water levels in a bay or estuary at remote locations without tide stations remain a challenge. Numerical hydrodynamic models are useful in simulating tidal currents in coastal areas with good accuracy (Wang et al., 2006), and the associated tidal signal with sea surface level can be predicted and simulated with relative ease (Li, Zhu & Zhang, 2016). Some researchers have used numerical hydrodynamic models to create a continuous spatial–temporal tidal level model that incorporates chart data to construct a more efficient instantaneous depth model (Brennan et al., 2007; Zhang et al., 2011; Zhang et al., 2010). However, these studies focused solely on constructing a high-precision tide model without considering the reference datum offset between static water depth and simulated tidal level or the deviation between simulated and measured water levels.

This article proposes a method for constructing a dynamic chart depth model at coastal areas, which is the sum of chart depths and dynamic water level models on the low water datum. The chart depths are obtained from the electronic navigational charts (ENCs). We focus on the accuracy of the dynamic water level model, which is the sum of the series of tidal levels and non-tidal components on the same reference datum. To simulate the series of tidal levels, we use the MIKE21 Flow Model. Validation of the tidal levels shows that the water levels deviation between the measured and simulated water levels at several discrete sites in the study area has a high spatial correlation. We propose a deviation correction method to mitigate the water levels deviation, which includes datum offset correction and residual water level correction. Two correction algorithms can reduce the water levels deviation and preserve the characteristics of the non-tidal distribution in the spatial field. Cross-validation is used to verify the deviation correction effect. The results indicate that the corrected dynamic water levels are close to the actual water levels. By merging the corrected dynamic water levels and the chart depth, we can obtain high-accuracy dynamic chart depth at a given time and place. During abnormal weather conditions, the dynamic water level of the route is predicted and broadcast to sailing ships to provide dynamic chart depth guarantee for navigation safety.

## Materials & Methods

This article presents a dynamic chart depth model designed to provide real-time depth information to mariners navigating in coastal waters. The model integrates chart depth and dynamic water levels, which encompass tidal levels and residual water levels measured against the same reference datum. To simulate tidal levels in the study region, we utilized the 2D MIKE21 Flow Model. To address the issue of datum offset and residual water level, we propose a deviation correction method. The dynamic chart depth model incorporates tidal levels, residual water levels, datum offset correction values, and chart depth.

### Tidal level simulation

This article presents a dynamic chart depth model that can provide real-time depth information to mariners navigating in the coastal ocean. The model takes into account chart depth as well as dynamic water levels, which include both tidal levels and residual water levels on the same reference datum. To simulate tidal levels in the study region, we used the 2D MIKE21 Flow Model. We also proposed a deviation correction method to mitigate the effect of datum offset and residual water level. Finally, we propose a dynamic chart depth model that integrates the tidal levels, residual water levels, datum offset correction values, and chart depth (Fadlillah, Widyastuti & Marfai, 2020; Warren & Bach, 1992; Lian et al., 2015).

In a simulation model, open boundary conditions and initial conditions play crucial roles in defining the model domain. Previous studies have suggested two methods for obtaining the dynamic water levels for the open boundary conditions. The first method involves extracting the global tidal models, while the second method involves interpolating the observed water levels near the open boundary (Cancet et al., 2018; Harker et al., 2019). However, the accuracy of water levels obtained from global tidal models is insufficient for high-precision simulations in shallow water regions (Li, Zhu & Zhang, 2016). The observed water levels consist of tidal levels and non-tidal level components. Unfortunately, due to the lack of meteorological forcing conditions, the MIKE21 Flow Model is unable to accurately simulate the non-tidal level component. Therefore, the tidal table is the only source of reliable data for obtaining the tidal levels for the open boundary conditions of the domain. In this study, the National Marine Data & Information Service of China provided tide tables consisting of 122 tidal constituents. We used the time-difference method to interpolate the water levels of the tide tables to each node of the open boundaries, respectively (Liu, Bao & Jun, 2019). The open boundary water level referenced to the low water datum. Assuming that the tidal levels of the starting points A and C of the two open boundaries are *H1* and *H* 2, respectively. The open boundary AB is divided into *L*_1_ sections at equal intervals, and the open boundary BC is divided into *L*_2_ sections at equal intervals, then the tidal level *h1*_*n*(*t*)_ at the nth point from A to B on the open boundary AB and the tidal level *h2*_*m*(*t*)_ at the point from C to B on the open boundary BC at time *t* are:

\begin{eqnarray*}\Delta {H}_{ \left( t \right) }& =H{2}_{ \left( t \right) }-H{1}_{ \left( t \right) } \end{eqnarray*}

(1) \begin{eqnarray*}h{1}_{n(t)}& =H{1}_{ \left( t \right) }+ \frac{n}{{L}_{1}+{L}_{2}} \Delta {H}_{ \left( t \right) }\end{eqnarray*}

\begin{eqnarray*}h{2}_{m(t)}& =H{2}_{ \left( t \right) }- \frac{m}{{L}_{1}+{L}_{2}} \Delta {H}_{ \left( t \right) }. \end{eqnarray*}

In this way, the dynamic tidal levels on two open boundaries are obtained.

The initial conditions were set by sourcing bathymetric data and the shoreline from the ENC (Electronic Navigational Chart) produced by the Department of Navigation Guarantee, Chinese People’s Liberation Army Navy Command. The spatial resolution of the bathymetric data is approximately 100 m. Both the open boundary water levels and the bathymetric data are referenced to the low water datum, while the shoreline is referenced to the mean high-water springs. To compensate for the missing bathymetry data in the nearshore area, ETOPO1 data was used. To avoid any compromise on the accuracy of the forced water level, the bathymetry based on the low water datum was converted to the bathymetry based on the mean sea surface water. The depth datum values (L values) were obtained from tide stations with a discrete distribution within the study area. The chart water depth data was then interpolated and corrected based on the discrete L values to obtain the bathymetric data based on the mean sea level.

### Deviation correction method

We designed a deviation correction method to mitigate the water levels deviation, which has two parts: datum offset correction and residual water level correction.

#### Datum offset correction

In simulating tidal levels, open boundary conditions play a crucial role in determining the accuracy of the results. Unfortunately, the conventional method of setting these conditions is unable to obtain actual tidal information at open boundary nodes, resulting in an inevitable error between the obtained and actual water levels. This error is constant at each open boundary point and is transferred to the entire domain during simulation, leading to a vertical deviation between the simulated tidal level and the actual measured value. Through verification, it has been found that the values of vertical deviations between discrete distribution verification points are consistent with each other, suggesting that the error conforms to the same variation trend in the domain, and can be reduced through interpolation. The harmonic constant datum method (HCDM) is currently used to obtain tidal datum. The datum offset correction can be derived by subtracting the observed tidal water level from the simulated tidal level during the same period (Eq. (2)). The study area’s triangular grids provide the coordinates of each grid node, from which the simulated tidal level is extracted, and the simulated tidal datum value at the grid node is obtained following the tidal datum definition algorithm. Finally, the tidal datum of the study area is obtained by interpolation fitting, and the mathematical equations for calculating the tidal datum are provided below.

\begin{eqnarray*}\Delta {L}_{i}={L}_{{P}_{i}}-{L}_{{M}_{i}}& \end{eqnarray*}

\begin{eqnarray*}L=(fH)_{{K}_{1}}\cos \nolimits {\varphi }_{{K}_{1}}+(fH)_{{K}_{2}}\cos \nolimits \left( 2{\varphi }_{{K}_{1}}+2{g}_{{K}_{1}}-18{0}^{\circ }-{g}_{{K}_{2}} \right) & \nonumber\\\displaystyle -\sqrt{{ \left( { \left( fH \right) }_{{M}_{2}} \right) }^{2}+{ \left( { \left( fH \right) }_{{o}_{1}} \right) }^{2}+2{ \left( fH \right) }_{{M}_{2}}{ \left( fH \right) }_{{O}_{1}}\cos \nolimits \left( {\varphi }_{{K}_{1}}+{g}_{{K}_{1}}+{g}_{{O}_{1}}-{g}_{{M}_{2}} \right) }& \end{eqnarray*}

\begin{eqnarray*}-\sqrt{{ \left( { \left( fH \right) }_{{S}_{2}} \right) }^{2}+{ \left( { \left( fH \right) }_{{P}_{1}} \right) }^{2}+2{ \left( fH \right) }_{{S}_{2}}{ \left( fH \right) }_{{P}_{1}}\cos \nolimits \left( {\varphi }_{{K}_{1}}+{g}_{{K}_{1}}+{g}_{{P}_{1}}-{g}_{{S}_{2}} \right) }& \nonumber\\\displaystyle -\sqrt{{ \left( { \left( fH \right) }_{{N}_{2}} \right) }^{2}+{ \left( { \left( fH \right) }_{{Q}_{1}} \right) }^{2}+2{ \left( fH \right) }_{{N}_{2}}{ \left( fH \right) }_{{Q}_{1}}\cos \nolimits \left( {\varphi }_{{K}_{1}}+{g}_{{K}_{1}}+{g}_{{Q}_{1}}-{g}_{{N}_{2}} \right) }+{ \left( fH \right) }_{{M}_{4}}& \end{eqnarray*}

(2) \begin{eqnarray*}\cos \nolimits {\varphi }_{{M}_{4}}+{ \left( fH \right) }_{{M}_{6}}\cos \nolimits {\varphi }_{{M}_{6}}+{ \left( fH \right) }_{M{S}_{4}}\cos \nolimits {\varphi }_{M{S}_{4}}+{H}_{{S}_{a}}\cos \nolimits {\varphi }_{{S}_{a}}+{H}_{{S}_{Sa}}\cos \nolimits {\varphi }_{{S}_{Sa}}& \end{eqnarray*}

\begin{eqnarray*}{\varphi }_{{M}_{4}}=2{\varphi }_{{M}_{2}}+2{g}_{{M}_{2}}-{g}_{{M}_{4}}, \left( \textdegree \right) ;{\varphi }_{{M}_{6}}=3{\varphi }_{{M}_{2}}+3{g}_{{M}_{2}}-{g}_{{M}_{6}}, \left( \textdegree \right) ;& \end{eqnarray*}

\begin{eqnarray*}{\varphi }_{M{S}_{4}}={\varphi }_{{M}_{2}}+{\varphi }_{{S}_{2}}+{g}_{{M}_{2}}+{g}_{{S}_{2}}-{g}_{M{S}_{4}}, \left( \textdegree \right) ;& \end{eqnarray*}

\begin{eqnarray*}{\varphi }_{{M}_{2}}=t{g}^{-1} \left( \frac{{ \left( fH \right) }_{{o}_{1}}\sin \nolimits \left( {\varphi }_{{K}_{1}}+{g}_{{K}_{1}}+{g}_{{O}_{1}}-{g}_{{M}_{2}} \right) }{{ \left( fH \right) }_{{M}_{2}}+{ \left( fH \right) }_{{O}_{1}}\cos \nolimits \left( {\varphi }_{{K}_{1}}+{g}_{{K}_{1}}+{g}_{{O}_{1}}-{g}_{{M}_{2}} \right) } \right) +18{0}^{\circ }, \left( \textdegree \right) ;& \end{eqnarray*}

\begin{eqnarray*}{\varphi }_{{S}_{2}}=t{g}^{-1} \left( \frac{{ \left( fH \right) }_{{P}_{1}}\sin \nolimits \left( {\varphi }_{{K}_{1}}+{g}_{{K}_{1}}+{g}_{{P}_{1}}-{g}_{{S}_{2}} \right) }{{ \left( fH \right) }_{{S}_{2}}+{ \left( fH \right) }_{{P}_{1}}\cos \nolimits \left( {\varphi }_{{K}_{1}}+{g}_{{K}_{1}}+{g}_{{P}_{1}}-{g}_{{S}_{2}} \right) } \right) +18{0}^{\circ }, \left( \textdegree \right) ;& \end{eqnarray*}

\begin{eqnarray*}{\varphi }_{{S}_{a}}={\varphi }_{{K}_{1}}- \frac{1}{2} {}_{2}+{g}_{{K}_{1}}- \frac{1}{2} {g}_{{S}_{2}}-18{0}^{\circ }-{g}_{{S}_{a}}, \left( \textdegree \right) ; \end{eqnarray*}

\begin{eqnarray*}{\varphi }_{{S}_{Sa}}=2{\varphi }_{{K}_{1}}-{}_{2}+2{g}_{{K}_{1}}-{g}_{{S}_{2}}-{g}_{{S}_{Sa}}, \left( \textdegree \right) ;& \end{eqnarray*}

\begin{eqnarray*}{}_{2}={\varphi }_{{S}_{2}}-18{0}^{\circ }, \left( \textdegree \right) ;& \end{eqnarray*}

where *Q*_1_, *O*_1_, *P*_1_, *K*_1_, *N*_2_, *M*_2_, *S*_2_, *K*_2_, *M*_4_, *MS*_4_, *M*_6_, *S*_*a*_ and *S*_*Sa*_ are partial tides, expressed by the amplitude and phase (the so-called tidal harmonic constants). The datum offset is obtained by HCDM of the observed water level and simulated water level at the same location in the same time periods, and then calculated by Eq. (2). The parameters of the Eq. (2) are explained in Table 1.

**Table 1:** The values of parameter in the Eq. (1).

**Parameters**	**Values**
*L*	The value of datum, which is the height of the depth datum below mean sea level.
Δ*L*_*i*_	The value of datum deviation at gauging station *i*, which is the datum deviation between datum value of predicted water level with simulated water level.
*L*_*P*_*i*__	The datum value of predicted water level at gauging station *i*.
*L*_*M*_*i*__	The datum value of simulated water level at gauging station *i*.
*f*	The nodal variations of the partial tides.
*H*	The amplitude of the partial tides, usually given in feet or meters.
*g*	The epoch of partial tide, usually expressed in degrees.
*φ*	The phase angle, the range is 0–360° .

In situations where the values of datum offset correction at discrete verification points exhibit proximity to each other, the inverse distance weighting (IDW) interpolation algorithm is a frequently employed technique to ameliorate the datum offset in the surveyed region. The mathematical formula for this algorithm is as follows:

(3) \begin{eqnarray*}{L}_{j}=\sum _{i}^{n}{\lambda }_{ij}\cdot [-{L}_{i}],{\lambda }_{ij}={l}_{ji}^{-p} \left/ \right. \sum _{i=1}^{n}{l}_{ji}^{-p}\end{eqnarray*}

where *L*_*j*_ is the revised value of the datum offset at depth point *j*; *L*_*i*_ is the corrected value for the datum offset at gauging station *i*; *λ*_*ij*_ is the weight of gauging station *i* at point *j*; *n* is the number of tide stations; *i* is the gauging station number; *l*_*ji*_ is the straight-line distance between station *i* and point *j*; and *p* is the index of the weighting function, and the default value is 2.

#### Residual water level correction

The residual water level is determined by meteorologically induced changes in sea level, including storm surge heights, local wind set-up, and the inverted barometer effect (Ningsih, Suryo & Anugrah, 2011). Due to the intricate nature of meteorological disturbances, modeling this phenomenon can be challenging.

To calculate the residual water level, the tidal level distribution must be removed from the measured water level. The following mathematical equation illustrates how to compute the residual water level:

(4) \begin{eqnarray*}{R}_{i} \left( t \right) ={H}_{i} \left( t \right) -{h}_{0}-\sum _{k=1}^{m}{f}_{k}{h}_{k}\cos \nolimits ({\sigma }_{k}t+{\upsilon }_{k}+{u}_{k}-{g}_{k})\end{eqnarray*}

where *R*_*i*_(*t*) is the residual water level of gauging station *k* at time *t* ; ${H}_{i} \left( t \right) $ is the measured water level of gauging station *i* at time *t*, and the time interval is usually less than an hour; and *h*_0_ is the mean sea level, which is usually replaced by the calculated mean water level. *h*_*k*_ and *g*_*k*_ are the amplitude and phase, respectively; *m* is the number of partial tides after analyzing the measured water level; *f*_*k*_ and *u*_*k*_ are a nodal factor and nodal angle; *σ*_*k*_ is the angular rate; and *υ*_*k*_ is the initial phase of the astronomic constituent. The accuracy of the residual water level extraction relies heavily on the precision of the tide harmonic analysis in the measured water level. Due to the ocean water level’s same or similar inducement and inertia, the residual water level demonstrates short-term and local regularity, resulting in strong spatial correlation (Tang, Bao & Jun, 2007; Zhao et al., 2017). The distance weighted interpolation algorithm is widely employed in meteorology (Degaetano & Belcher, 2006) to create a two-dimensional field from finite observational data using a range-dependent weighting function. We improved the interpolation algorithm by considering both distance and correlation factors to reconstruct the residual water level field in the survey region. To obtain the weight value of each site during interpolation, we compared the residual water levels between stations and calculated the mean correlation coefficient between the residual water level and other stations. This mean value is used as the weight value of the site.

\begin{eqnarray*}{R}_{j} \left( t \right) & =\sum _{i=1}^{n}{\mu }_{i}\cdot \left[ -{R}_{i} \left( t \right) \right] \cdot {\lambda }_{ij},{\mu }_{i}= \frac{n\cdot \sum _{k=1}^{n-1}{\rho }_{ik}}{\sum _{i=1}^{n}\sum _{k=1}^{n-1}{\rho }_{ik}} \end{eqnarray*}

(5) \begin{eqnarray*}{\rho }_{ik}& = \frac{\sum _{t}^{N}({R}_{i} \left( t \right) -\overline{{R}_{i} \left( t \right) })({R}_{ki} \left( t \right) -\overline{{R}_{k} \left( t \right) })}{\sqrt{\sum _{t}^{N}({R}_{i} \left( t \right) -\overline{{R}_{i} \left( t \right) })^{2}\sum _{t}^{N}({R}_{k} \left( t \right) -\overline{{R}_{k} \left( t \right) })^{2}}} \end{eqnarray*}

where ${R}_{j} \left( t \right) $ is the residual water level at point *j* at time *t*; *μ*_*i*_ is the correlation coefficient value of tide station *i*; ${R}_{i} \left( t \right) $ is the residual water level of tide station *i* at time *t*; *λ*_*ij*_ is the weight of gauging station *i* at point *j*; *ρ*_*ik*_ is the simulation deviation correlation coefficient of tide station *i* with station $k.{R}_{k} \left( t \right) $ is the residual water level of tide station *k* at time *t*; and *N* is the number of residual water level records.

### Dynamic chart depth

We propose a dynamic water level model that integrates the tide level, residual water level, datum offset correction value, and chart depth. To simulate the tide, we created a triangulated mesh grid. At each grid point, the tide can be extracted and deviation/tidal components and chart depth can be interpolated using a suitable algorithm. This allows us to construct the instantaneous water level at any given grid point, as follows:

(6) \begin{eqnarray*}{D}_{j} \left( t \right) ={h}_{j} \left( t \right) +{R}_{j} \left( t \right) +{L}_{j}+{d}_{j}\end{eqnarray*}

where ${D}_{j} \left( t \right) $ is the instantaneous water depth at point j in time *t*; *h*_*j*_(*t*) is the simulated tidal level at point j in time *t*; ${R}_{j} \left( t \right) $ is the residual water level correction at point j in time *t*; ${L}_{j} \left( t \right) $ is the datum offset correction value at point j; and *d*_*j*_ is the chart depth at point j.

## Results

### Tide simulation and validation

#### Study area and model setting

**Figure 1:**
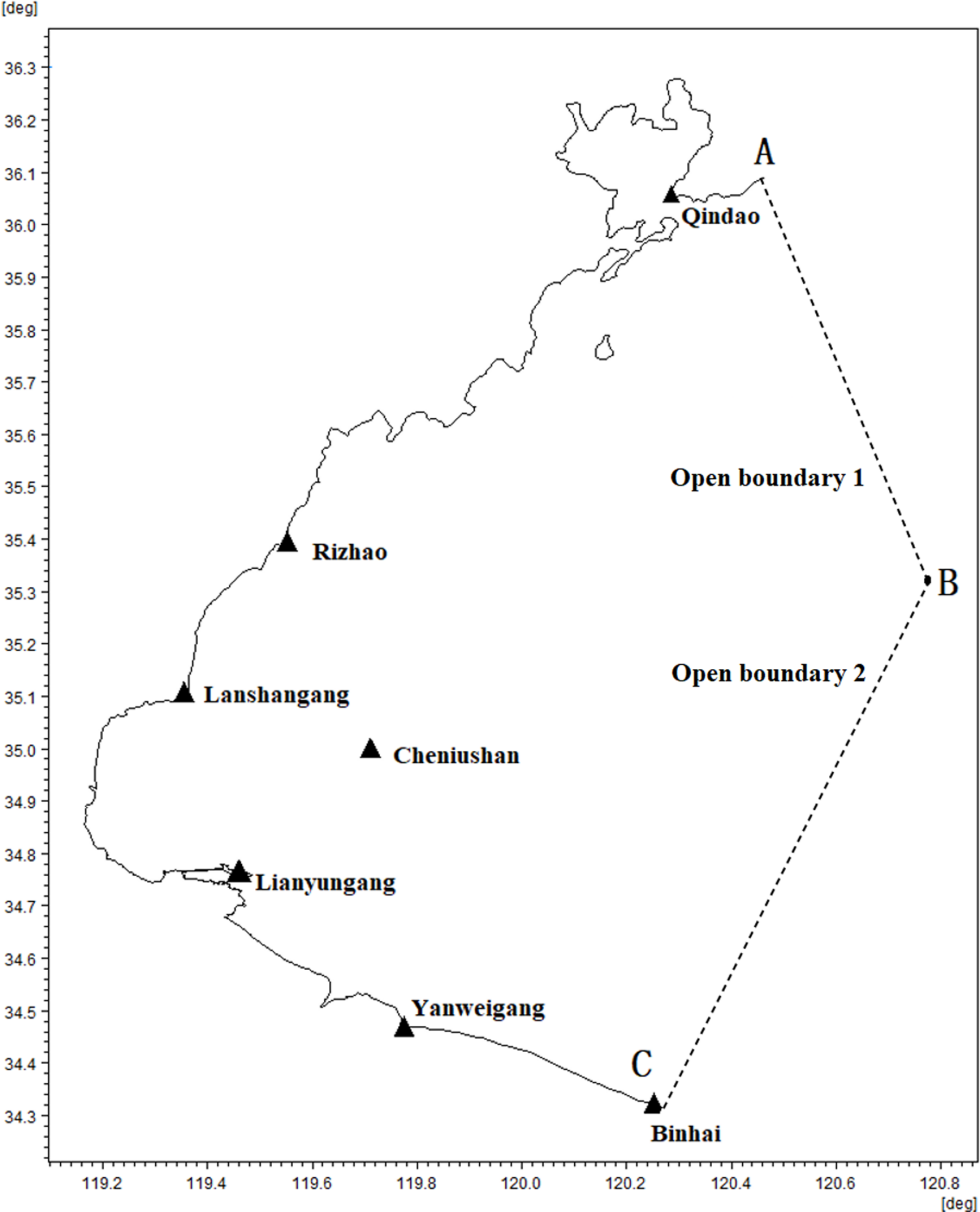
Location of tide gauge stations and open boundaries. Black triangles represent tide stations and broken lines represent open boundaries.

In this study, we developed a numerical hydrodynamic model using the MIKE21 Flow Model to simulate the tide in the southwest part of the Yellow Sea within the domain of 118°42′∼120°47′ longitude and 34°32′∼36°28′ latitude. The study area includes seven tide stations, six of which are along the coast and one on Cheniushan Island (see Fig. 1 and Table 2). We corrected the chart depth using the seven discrete L values obtained from the tide station distribution and derived the bathymetric data based on the mean sea level. Considering the features of the study area, we established two open boundaries that start from the tide survey stations and run perpendicular to the coastline before converging at a certain point in the sea. To specify the open boundary conditions, we used the tide tables to provide the dynamic tide levels. Using the Time-Difference Method, we interpolated the water levels of the two tide tables to each node of the open boundaries. Both the open boundary water level and bathymetric data were referenced to the low water datum.

**Table 2:** Tidal station information.

**Station name**	**Lon.**	**Lat.**	**Type**	**Duration**
Rizhao	119.55	35.3667	Tide table	2007/01/01–2007/12/31
Cheniushan	119.8221	34.99513	Measurement	2007/01/16–2007/02/15
Qindao	120.3	36.083	Tide table	2007/01/01–2007/12/31
Yanweigang	119.8	34.5	Tide table	2007/01/01–2007/12/31
Lanshangang	119.3753	35.0863	Measurement	2007/01/16–2007/02/15
Lanshangang	119.3753	35.0863	Tide table	2007/01/01–2007/12/31
Lianyungang	119.4648	34.74942	Measurement	2007/01/16–2007/02/15
Lianyungang	119.4648	34.74942	Tide table	2007/01/01–2007/12/31
Kaishandao	119.8713	34.52968	Measurement	2007/01/16–2007/02/15
Binhai	120.267	34.302	Tide table	2007/01/01–2007/12/31

The method for setting the domain of this model is based on Tian et al.’s (2023) method. To simulate the dynamic boundary of the intertidal zone during tidal rise and fall, we employed the wet/dry point treatment method. The temperature and salinity of the model were set to constant values of 10 °C and 0.035, respectively. The model’s time step interval was set at 0–10 s, the output data frequency was one hour, and the simulation period was from January 1st, 2007, to March 31st, 2007. To set the initial and boundary conditions, we collected reliable data from ENCs as described in Tian et al. (2023). The shoreline data was referenced to the Mean High Water Springs benchmark, and the bathymetric data was referenced to the low tide datum.

**Figure 2:**
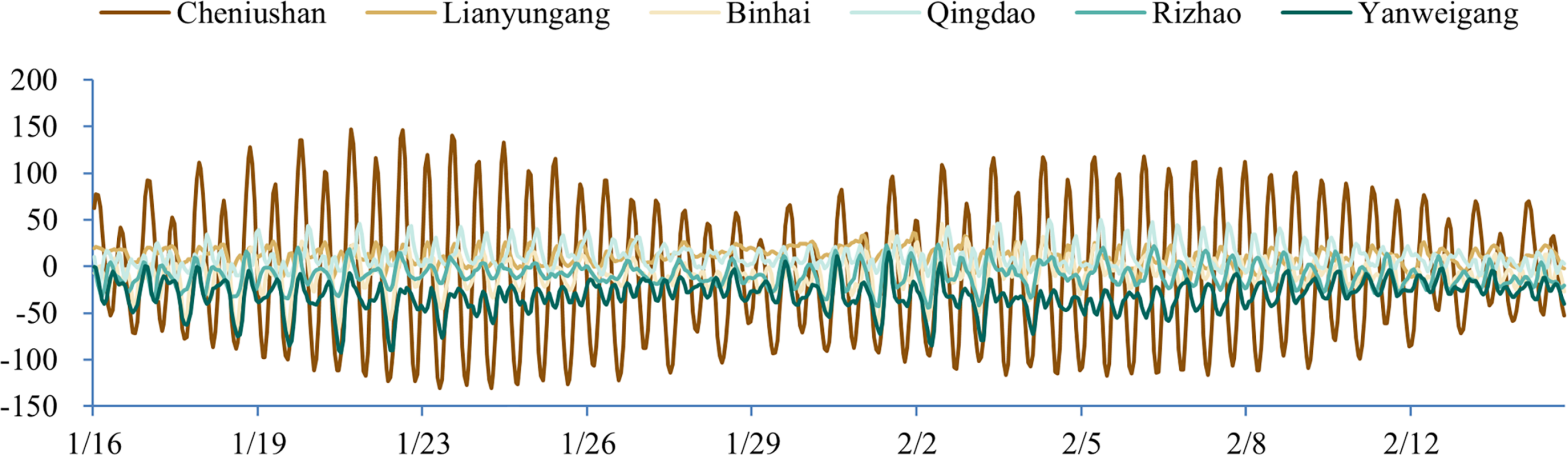
Accuracy comparison between tide table and DTU10 global tide model (unit: cm).

This study utilized tidal tables to provide dynamic tidal levels for open boundaries instead of relying on global tidal models, such as DTU10, due to their low accuracy nearshore. In contrast, the astronomical tide predicted by the tide tables offers high accuracy. We compared the accuracy of the tidal tables and the global tidal model using measured data from several tidal stations. The tidal stations located near the shore (Lianyungang, Binhai, Qingdao, Rizhao, and Yanweigang) were evaluated by comparing the tide table with the astronomical tide predicted by the measured data. For the tidal stations located in the sea (Cheniushan), we compared the DTU10 global tide model with the astronomical tide predicted by the measured data. The specific results are presented in Fig. 2.

As shown in Fig. 2, the tide table provided a more accurate estimate of the actual measured water level than the DTU10 global tide model, making it the preferred option to provide open boundary conditions in this study.

#### Deviation corrections and verification

**Figure 3:**
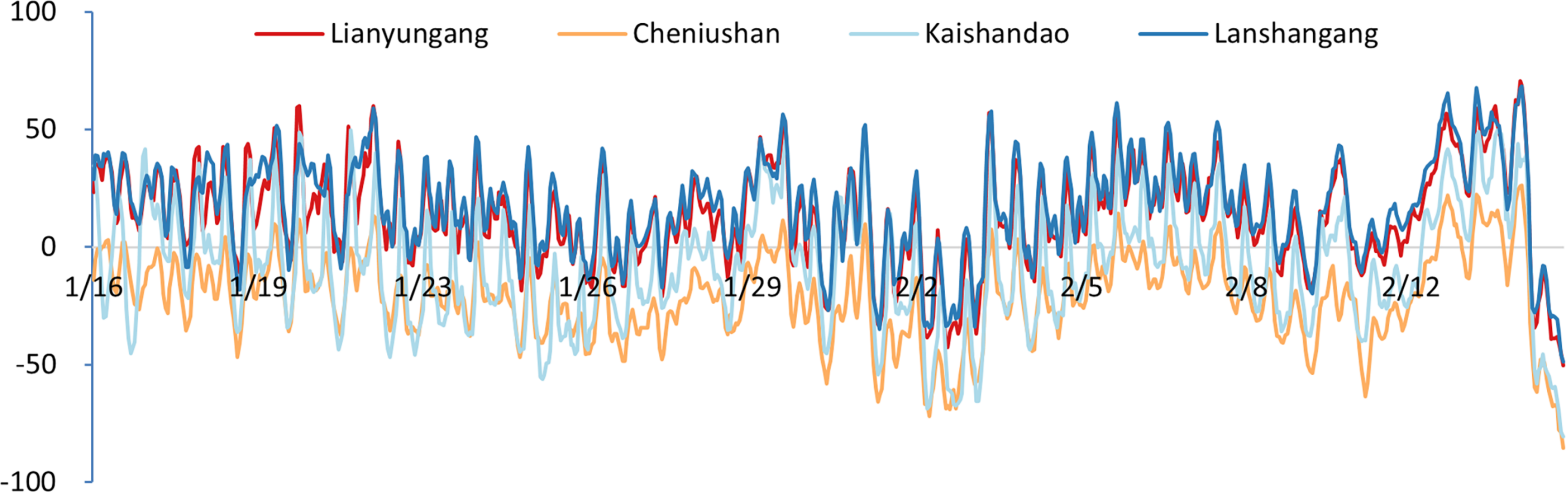
Time-series plots of the simulated deviations at the four gauging stations (unit: cm).

**Figure 4:**
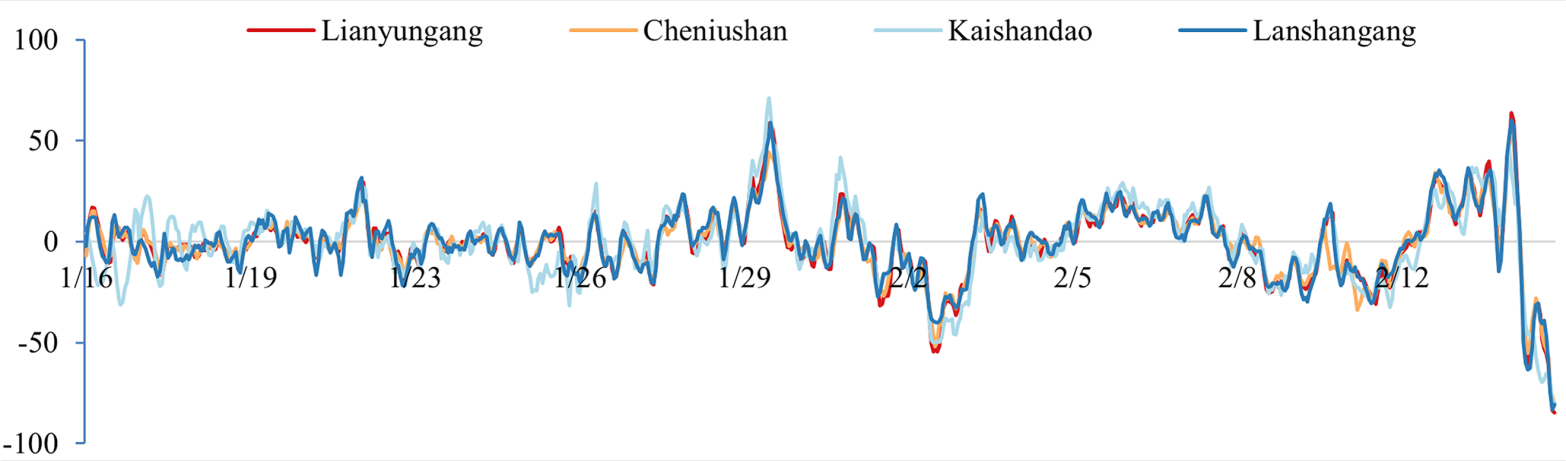
Time-series plots of the residual water level at the four gauging stations (units: cm).

**Figure 5:**
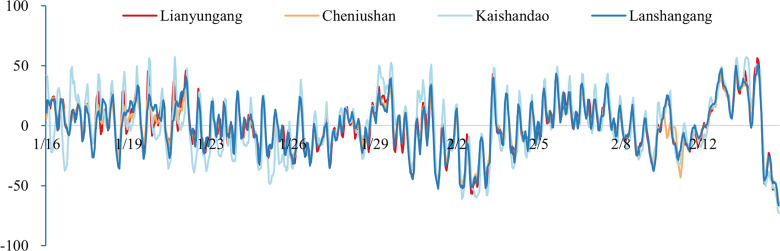
The time-series plots of the water level deviation of the four gauging stations after datum deviation correction (unit: cm).

To validate the model results, we compared the simulated tidal water level with the observed tidal gauge data from the survey region. As indicated in Table 3 and Fig. 3, the water level deviations from several verification stations in the study area exhibit similar time-series variations, suggesting an inherent temporal characteristic of the study area. Consequently, we applied the deviation correction method to mitigate these deviations. The datum offsets of the four stations were determined using Eq. (2) and presented in Table 4. The residual water levels of the four tide stations calculated using Eq. (4) are displayed in Fig. 4, and the correlation coefficients for each station are provided in Table 5. We first corrected the datum offset for the survey region using Eq. (3), and the results are shown in Fig. 5 and Table 6.

**Table 3:** The observed and simulated values of tide at tide station and the water level deviation between them, and the vertical datum is low water datum (unit: cm).

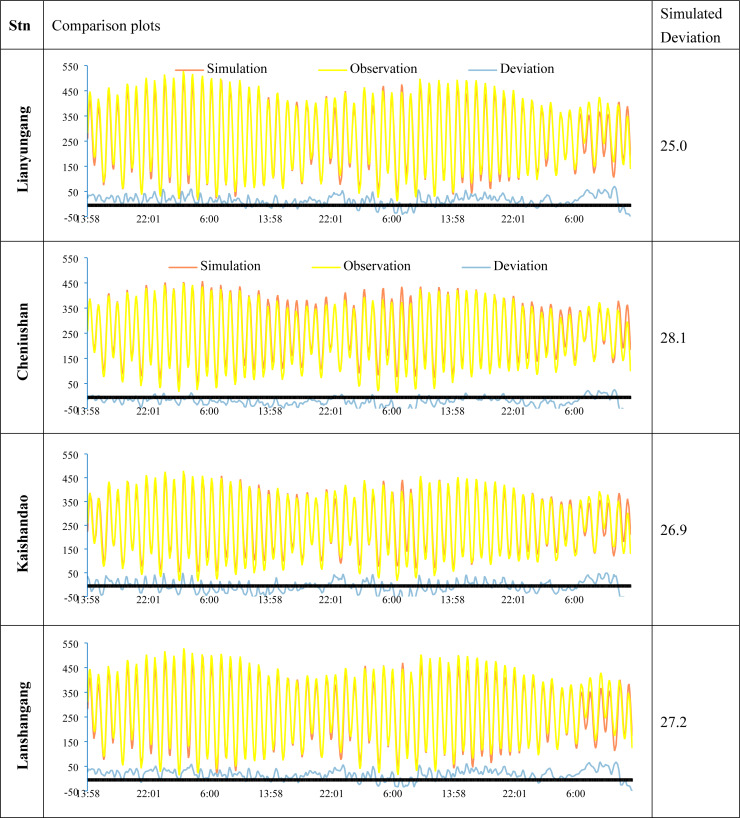

**Table 4:** The datum deviation values of the four tide stations (unit: cm).

**Lianyungang**	**Cheniushan**	**Kaishandao**	**Lanshangang**
13.7	15.1	25.3	18.5

**Table 5:** Residual water level correlation coefficients of the four stations from January 16th, 2007, to January 31st, 2007.

	**Lianyungang**	**Cheniushan**	**Kaishandao**	**Lanshangang**
Lianyungang	1			
Cheniushan	0.971893	1		
Kaishandao	0.895002	0.882379	1	
Lanshangang	0.970974	0.959983	0.868586	1

**Table 6:** Root-mean-square deviation of the water level deviation after datum deviation correction (unit: cm).

**Lianyungang**	**Cheniushan**	**Kaishandao**	**Lanshangang**
20.9	18.5	25.6	20.8

**Figure 6:**
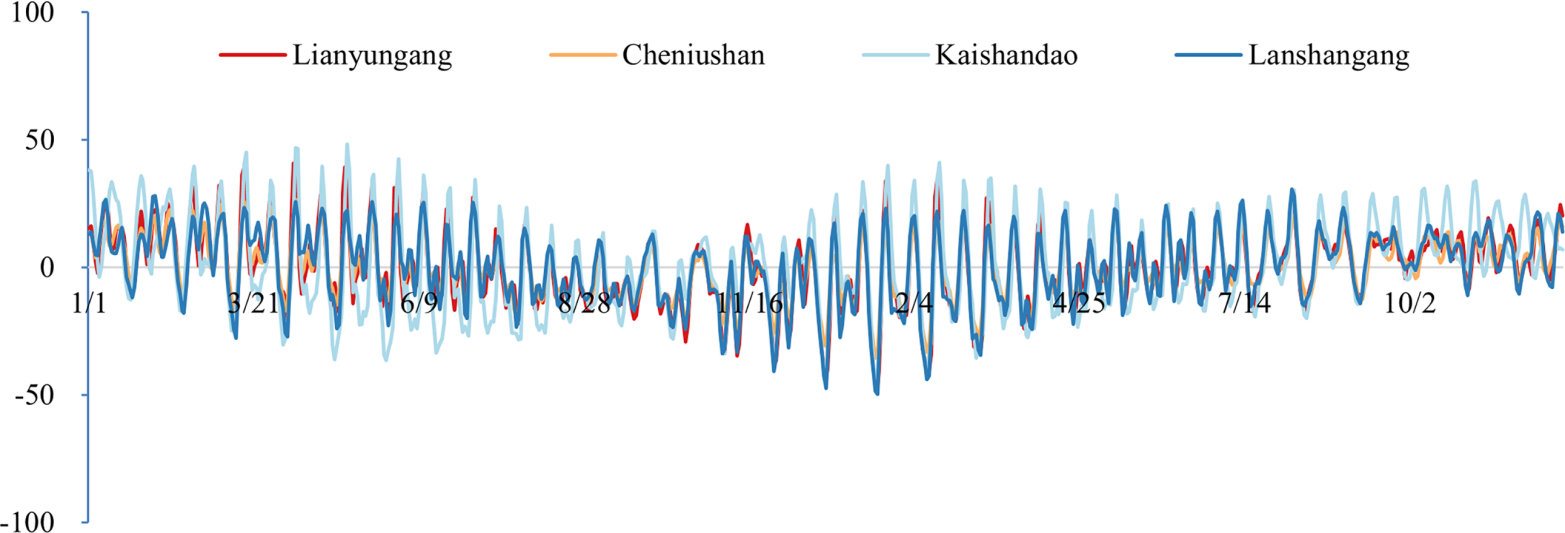
The time-series plots of the water level deviations of the four gauging stations after datum deviation correction and residual water level correction (unit: cm).

After correcting the datum offsets, we reconstructed the residual water level field using Eq. (5) and compared the corrected and uncorrected results, as illustrated in Fig. 6 and Table 7.

**Table 7:** Root-mean-square deviation of the water level deviation after datum deviation correction and residual water level correction (unit: cm).

**Lianyungang**	**Cheniushan**	**Kaishandao**	**Lanshangang**
14.4	11.0	18.2	14.2

 To verify the accuracy of the corrected instantaneous water level in the survey region, we designed a cross-validation method. We selected one of the four tidal stations as the reference point and used the water levels from the other three stations to calculate the instantaneous water level of the selected station. We then compared the results with the actual water level by calculating the root-mean-square deviation.

As indicated in Tables 7 and 8, the results of the two corrections are similar, demonstrating the applicability of the proposed deviation correction method.

**Table 8:** Root-mean-square deviation of the corrected results of each station compared with the actual observation (unit: cm).

**Lianyungang**	**Cheniushan**	**Kaishandao**	**Lanshangang**
13.7	13.3	21.3	13.2

### Results of the dynamic chart depth model

**Figure 7:**
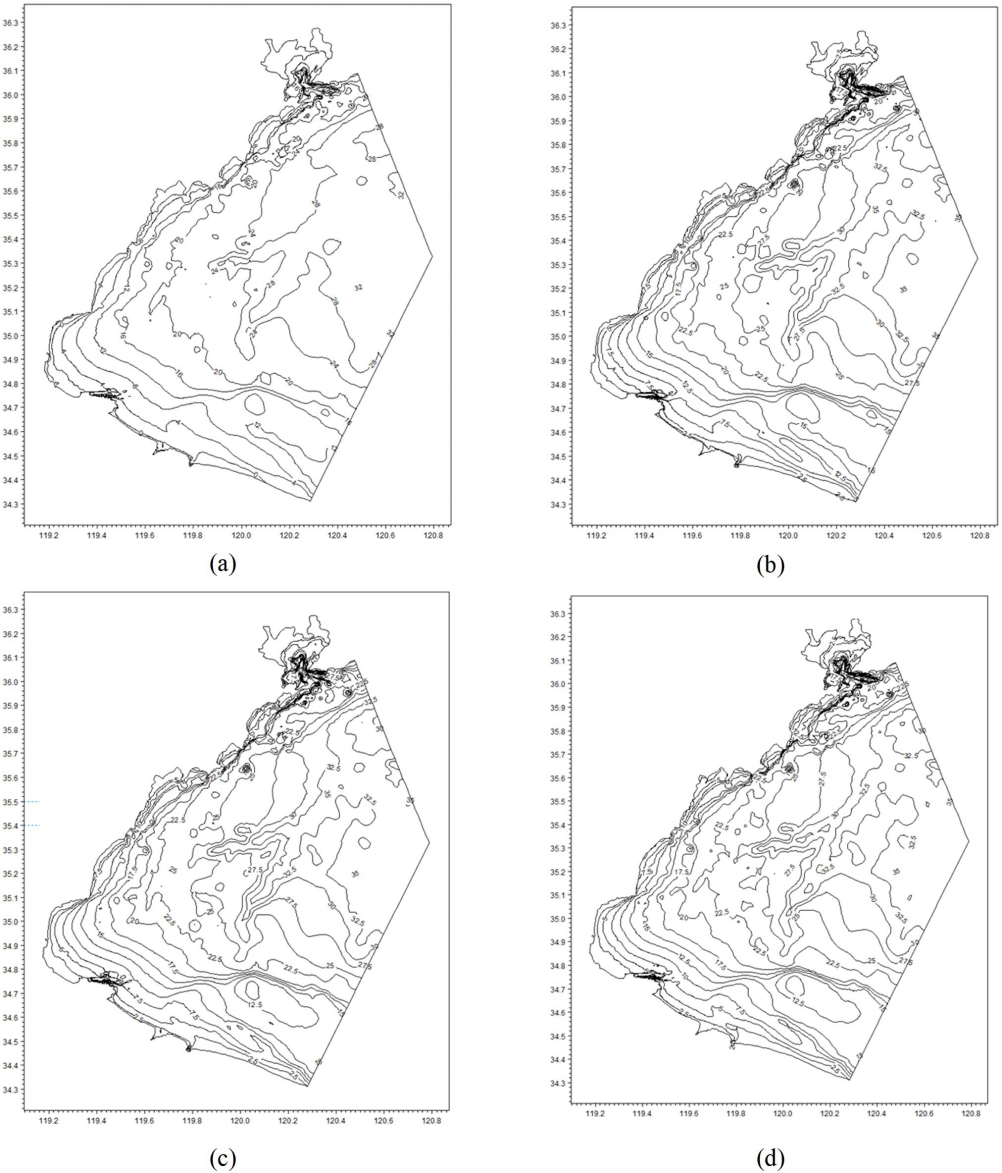
The bathymetry at different times: (A) is the static nautical chart which is a below-water topographic map; (B) is the lowest depth chart at 15:00 on 2007-2-12; (C) is the mean depth chart at 11:00 on 2007-2-12; and (D) is the highest depth chart.

After correcting the water level deviation component, we were able to determine the instantaneous water depth at any point in the region at any given time using Eq. (6). To illustrate the results of the dynamic chart depth model, we compared the lowest, mean, and highest water depths of the region with the nautical chart depth. As shown in Fig. 7, there are some discrepancies between the isobaths of the four depth charts. These differences are primarily due to variations in the range and timing of the tide, as well as differences in the amplitude and timing of the dynamic water level changes in the region. Additionally, it is worth noting that even the lowest depth chart is still deeper than the nautical chart. Therefore, the dynamic chart depth exceeds the nautical chart depth.

## Discussion

The datum offset is mainly caused by the open boundary water level setting since it is difficult to obtain the actual water level of the boundary. Therefore, interpolated water level errors are inevitable. In this study, we obtained the tidal datum from water level records and the water level simulated during the same period using HCDM. This method produces a more accurate and stable datum offset compared to calculating the mean water level directly. Additionally, it avoids the effect of non-tidal components.

The residual water level presents short-term and local regularity due to the same or similar inducement and inertia of ocean water level body movement. The local regularity arises from the strong spatial correlation of the residual water level. The spatial gradient of the residual water level is higher than that of the tide in both offshore and coastal waters because of this strong correlation (Guan et al., 2019). An appropriate interpolation algorithm can be used to reconstruct the regional residual water level field based on the residual water level sequence with reasonable discrete points (Hess, 2003; Tang, Bao & Jun, 2007). The widely used distance weighted interpolation algorithm is employed in this study. However, the similarity coefficient of the residual water level sequence between tide stations must also be considered. The station with the highest similarity should have the largest weight in interpolation. To address this issue, an improved distance weighted interpolation algorithm is used in this article, which takes into account the effect of similar variable relations of the residual water level in the spatial field and the weight representation of each tide station residual water level sequence.

## Conclusions

The safety of a ship’s route depends on the sea depth being equal to or greater than the safety depth. However, because of tidal action, the water depth is always changing. To compensate for the limitations of tidal prediction technology, the safety depth is set below the tidal datum to prevent the effect of tidal changes. This causes the charted safe navigation area to be smaller than the actual area. In this study, we propose a method for constructing a dynamic chart depth model for a coastal region by combining the static chart depth and dynamic water levels on the same reference datum. Our findings suggest that:

(1) A deviation correction method is needed to mitigate the water level deviations. The algorithm can eliminate similar variable water level deviations and recover the actual tide heights in the spatial field. We verified the results of the tide model and discovered a similar variable relation between the partial tide components in the spatial field of several of the discrete verification stations in the study area.

(2) The actual safety depths are always changing due to differences in the amplitude and time of the tide. This article provides a modeling method for the actual safety depth, which could increase the nautical time and range of ships. Additionally, it provides highly accurate instantaneous tide levels and depth information for use in coastal engineering.

##  Supplemental Information

10.7717/peerj.15616/supp-1 Supplemental Information 1 Raw data

## Additional Information and Declarations

### Data Availability

The following information was supplied regarding data availability: The raw data is available in the Supplementary File.
